# The PaO_2_/FiO_2_ is independently associated with 28-day mortality in patients with sepsis: a retrospective analysis from MIMIC-IV database

**DOI:** 10.1186/s12890-023-02491-8

**Published:** 2023-05-27

**Authors:** Hongying Bi, Xu Liu, Chi Chen, Lu Chen, Xian Liu, Jianmin Zhong, Yan Tang

**Affiliations:** 1 Department of Critical Care Medicine, The Affiliated Hospital of Guizhou Medical University, Guiyang, Guizhou China; 2 Department of Immunology and Microbiology, Guiyang College of Traditional Chinese Medicine, Guiyang, Guizhou China; 3 Clinical Trials Centre, The Affiliated Hospital of Guizhou Medical University, Guiyang, Guizhou China; 4 Guizhou Medical University, Guiyang, Guizhou, China

**Keywords:** Sepsis, 28-day mortality, PaO_2_/FiO_2_, MIMIC-IV database

## Abstract

**Background:**

To clarify the relationship between the PaO_2_/FiO_2_ and 28-day mortality in patients with sepsis.

**Methods:**

This was a retrospective cohort study regarding MIMIC-IV database. Nineteen thousand two hundred thirty-three patients with sepsis were included in the final analysis. PaO_2_/FiO_2_ was exposure variable, 28-day mortality was outcome variable. PaO_2_/FiO_2_ was log-transformed as LnPaO_2_/FiO_2_. Binary logistic regression was used to explore the independent effects of LnPaO_2_/FiO_2_ on 28-day mortality using non-adjusted and multivariate-adjusted models. A generalized additive model (GAM) and smoothed curve fitting was used to investigate the non-linear relationship between LnPaO_2_/FiO_2_ and 28-day mortality. A two-piecewise linear model was used to calculate the OR and 95% CI on either side of the inflection point.

**Results:**

The relationship between LnPaO_2_/FiO_2_ and risk of 28-day death in sepsis patients was U-shape. The inflection point of LnPaO_2_/FiO_2_ was 5.30 (95%CI: 5.21—5.39), which indicated the inflection point of PaO_2_/FiO_2_ was 200.33 mmHg (95%CI: 183.09 mmHg—219.20 mmHg). On the left of inflection point, LnPaO_2_/FiO_2_ was negatively correlated with 28-day mortality (OR: 0.37, 95%CI: 0.32—0.43, *p* < 0.0001). On the right of inflection point, LnPaO_2_/FiO_2_ was positively correlated with 28-day mortality in patients with sepsis (OR: 1.53, 95%CI: 1.31—1.80, *p* < 0.0001).

**Conclusions:**

In patients with sepsis, either a high or low PaO_2_/FiO_2_ was associated with an increased risk of 28-day mortality. In the range of 183.09 mmHg to 219.20 mmHg, PaO_2_/FiO_2_ was associated with a lower risk of 28-day death in patients with sepsis.

## Introduction

Sepsis is defined as a potentially fatal organ dysfunction caused by dysregulated host response to infection [1]. In 2017, an estimated 489 million incident cases of sepsis were recorded worldwide, with 110 million sepsis-related deaths reported, accounting for 19.7% of global mortality [2]. Furthermore, sepsis has become the leading cause of intensive care unit (ICU) admission and in-hospital death [2–4]. Early identification and appropriate management of sepsis may improve patient survival outcomes [1]. To find out the possible cause of death of septic patients may be important for sepsis management.

Recently, the arterial partial pressure of oxygen (PaO_2_) has attracted much attention. As we know, low PaO_2_ is associated with high mortality in critically ill patients [5, 6]. However, although higher PaO_2_ levels may improve the oxygen delivery, they can also lead to potential harm such as the tissue injury. To correct the effect of FiO_2_ on the PaO_2_, the ratio of arterial partial pressure of oxygen (PaO_2_) to fraction of inspired oxygen (FiO_2_) is usually used. PaO_2_/FiO_2_ is important for the diagnosis of sepsis as one of the variables in sequential organ failure assessment (SOFA) score [7, 8]. A retrospective study of 135 elderly patients with sepsis showed that PaO_2_/FiO_2_ was a promising tool and biomarker for predicting 28-day mortality [9]. High PaO_2_/FiO_2_ was an independent risk factor for 28-day mortality in patients with sepsis-related myocardial injury [10]. In addition, a novel blended machine learning (ML) model for hospital mortality prediction in ICU patients with sepsis identified the minimum PaO_2_/FiO_2_ as one of the top important predictors [11]. However, novel machine learning techniques are time-consuming to implement in practice, and are inapplicable to clinical work. Additional concerns are also raised about the clinical utility of results from studies with small sample sizes, population limitations, and failure to consider the possibility of non-linear relationships. More importantly, it is still unknown which range of PaO_2_ was appropriate in patients with sepsis.

We hypothesized that an abnormal PaO_2_/FiO_2_ was associated with a high risk of 28-day mortality in patients with sepsis. Therefore, we aimed to investigate the relationship between the PaO_2_/FiO_2_ and 28-day mortality in patients with sepsis using a large-scale database.

## Methods

### Data source

This was a retrospective analysis based on the Medical Information Marketplace for Intensive Care IV (MIMIC-IV) database. It gathered clinical data on patients who admitted to Beth Israel Deaconess Medical Center (BIDMC) from 2008 to 2019 [12]. The database is free to download after completing an accredited course on their official website. One of the authors, Lu Chen, has completed the accredited course and was responsible for data extraction (Record ID: 50,668,217). Our study was performed in accordance with the reports of studies conducted using the observation routine collected health data (RECORD) [13].

### Study population

In total, 377, 207 adult patient records were found in the MIMIC-IV database. Sepsis was diagnosed according to sepsis-3 criteria [1], sepsis-relevant ICD-9 codes (99,591—99,592), or ICD-10 codes (R652, R6520 and R6521) [14, 15]. The outcome variable was death from any cause during 28-day after ICU admission, and PaO_2_/FiO_2_ was the exposure variable (recorded as a continuous variable). We extracted PaO_2_/FiO_2_ data at ICU admission. Patients with missing exposure variable information were excluded from this study. We collected demographic factors such as gender (male / female), age (years), ethnicity, Charlson Comorbidity Index, SOFA score, use of mechanical ventilation and renal replacement therapy (RRT), use of glucocorticoids (dexamethasone, methylprednisolone, cortisol), use of vasoactive drugs (dopamine, dobutamine, noradrenaline), use of intravenous immunoglobulin (IVIG), use of antibiotics (carbapenem, cephalosporins, penicillin, vancomycin), vital signs on admission include temperature, heart rate, respiratory rate and mean arterial pressure as main covariates. The selection of these covariates was primarily based on our clinical experience as well as literature [16–19].

### Missing data description

Patients with missing exposure and outcome information were removed. The missing covariates in this study were less than 5% (0—4.1%), therefore, multiple interpolation was not used to fill in the gaps. 

### Statistical analysis

Continuous variables were expressed as mean ± standard deviation (normal distribution) or median (quartile) (skewed distribution). Categorical variables were expressed in frequency or as a percentage. Since this was a cohort study, we divided the exposure variables into four quartiles, the distribution of patient baseline characteristics differed across quartiles. The one-way ANOVA (normal distribution), Kruskal–Wallis H (skewed distribution) test and chi-square tests (categorical variables) was used to determine any statistical difference among the means and proportions of the groups. Univariate binary logistic regression model was used to evaluate the associations between exposure and outcome. Both non-adjusted and multivariate-adjusted models were used. We explored the association between PaO_2_/FiO_2_ and 28-day mortality using univariate and multivariable binary logistic regression models. We log-transformed PaO_2_/FiO_2_ to LnPaO_2_/FiO_2_, due to its skewed distribution. During the data analysis, we present non-adjusted models (no covariates adjusted), minimally-adjusted models (adjusted for demographic factors only, Model I), fully-adjusted models (adjusted for all covariates presented in Table 1, Model II), and odds ratio values (OR) with 95% confidence intervals (CI). LnPaO_2_/FiO_2_ was transformed from a continuous variable to a categorical variable (quartile) for sensitivity analysis, and *P* for trend was calculated to see if the results were robust when LnPaO_2_/FiO_2_ was used as a continuous variable versus a categorical variable. Furthermore, we used Hosmer–Lemeshow Test to assess the goodness of fit of the above three models (non-adjusted, adjusted model I and adjusted model II) and reported Chi-square and *P* values (using R ResourceSelection-package and Hoslem. test Function). A non-linear relationship cannot be ruled out because LnPaO_2_/FiO_2_ is a continuous variable. Given the binary logistic regression model’s inability to handle non-linear associations, we observed the relationship between LnPaO_2_/FiO_2_ and 28-day mortality in patients with sepsis using a generalized additive model (GAM) and smoothed curve fitting. If there was a non-linear correlation, we used a recursive algorithm to calculate the inflection point value and 95% confidence interval (bootstrapping), and used a two-piecewise linear model to calculate the OR and 95% CI on either side of the inflection point. All the analyses were performed with the statistical software packages R (http://www.R-project.org, The R Foundation) and EmpowerStats (http://www.Empowerstats.com, X&Y Solutions, Inc, Boston, MA).* P* values less than 0.05 (two-sided) were considered statistically significant.

**Table 1:** Baseline characteristics of patients according to LnPaO_2_/FiO_2_ (*N* = 19, 233)

	Total	Q1(2.89–5.16)	Q2(5.16–5.51)	Q3(5.51–5.81)	Q4(5.81–7.80)	*p*-value
N	19,233	4803	4808	4791	4821	
Age at admission(years)	65.57 ± 15.65	65.87 ± 15.44	66.24 ± 14.46	66.47 ± 15.08	63.69 ± 17.32	< 0.001
Sex, n(%)						0.004
female	11,613(60.41%)	2855 (59.44%)	2992 (62.23%)	2922 (60.99%)	2844 (58.99%)	
male	7610(39.59%)	1948 (40.56%)	1816 (37.77%)	1869 (39.01%)	1977 (41.01%)	
White, n (%)	12,735(66.21%)	3171 (66.02%)	3286 (68.34%)	3212 (67.04%)	3066 (63.60%)	< 0.001
Charlson Comorbidity Index	5.75 ± 2.87	6.14 ± 2.91	5.86 ± 2.80	5.68 ± 2.72	5.34 ± 2.98	< 0.001
SOFA	7.71 ± 3.98	9.14 ± 4.14	8.28 ± 3.82	7.13 ± 3.71	6.29 ± 3.63	< 0.001
Vital signs						
Heart rate (bpm)	104.77 ± 24.27	108.74 ± 25.68	104.44 ± 23.49	102.96 ± 23.47	102.94 ± 23.90	< 0.001
Respiratory rate (bpm)	26.31 ± 9.96	29.45 ± 9.47	26.86 ± 9.52	25.08 ± 9.88	23.84 ± 10.07	< 0.001
Temperature (℃)	36.71 ± 1.42	36.93 ± 1.39	36.75 ± 1.43	36.62 ± 1.40	36.53 ± 1.44	< 0.001
MAP (mmHg)	76.45 ± 9.43	75.33 ± 9.86	75.74 ± 9.01	77.37 ± 9.13	77.37 ± 9.52	< 0.001
FiO_2_	0.56 ± 0.22	0.72 ± 0.22	0.58 ± 0.20	0.50 ± 0.18	0.44 ± 0.18	< 0.001
PaO_2_ (mmHg)	97.85 ± 54.49	62.73 ± 19.71	82.42 ± 27.10	104.21 ± 40.62	141.92 ± 75.04	< 0.001
MV, n (%)						< 0.001
No	4940(25.70%)	1733 (36.08%)	1115 (23.19%)	1070 (22.33%)	1022 (21.20%)	
Yes	14,283(74.30%)	3070 (63.92%)	3693 (76.81%)	3721 (77.67%)	3799 (78.80%)	
RRT						< 0.001
No	17,885(92.88%)	4321 (89.96%)	4443 (92.41%)	4521 (94.36%)	4570 (94.79%)	
Yes	1368(7.12%)	482 (10.04%)	365 (7.59%)	270 (5.64%)	251 (5.21%)	
Noradrenaline						< 0.001
No	11,973(62.28%)	2501 (52.07%)	2929 (60.92%)	3180 (66.37%)	3363 (69.76%)	
Yes	7250(37.72%)	2302 (47.93%)	1879 (39.08%)	1611 (33.63%)	1458 (30.24%)	
Dopamine, n (%)						< 0.001
No	17,813(92.67%)	4355 (90.67%)	4439 (92.33%)	4466 (93.22%)	4553 (94.44%)	
Yes	1410(7.33%)	448 (9.33%)	369 (7.67%)	325 (6.78%)	268 (5.56%)	
Dobutamine, n (%)						0.02
No	18,384(95.63%)	4589 (95.54%)	4568 (95.01%)	4584 (95.68%)	4643 (96.31%)	
Yes	839(4.37%)	214 (4.46%)	240 (4.99%)	207 (4.32%)	178 (3.69%)	
IVIG, n (%)						< 0.001
No	18,810(97.85%)	4660 (97.02%)	4710 (97.96%)	4702 (98.14%)	4738 (98.28%)	
Yes	413(2.15%)	143 (2.98%)	98 (2.04%)	89 (1.86%)	83 (1.72%)	
Dexamethasone, n(%)						< 0.001
No	17,425(90.65%)	4336 (90.28%)	4410 (91.72%)	4403 (91.90%)	4276 (88.70%)	
Yes	1798(9.35%)	467 (9.72%)	398 (8.28%)	388 (8.10%)	545 (11.30%)	
Methylprednisolone, n (%)						< 0.001
No	15,887(82.65%)	3710 (77.24%)	3963 (82.43%)	4077 (85.10%)	4137 (85.81%)	
Yes	3336(17.35%)	1093 (22.76%)	845 (17.57%)	714 (14.90%)	684 (14.19%)	
Cortisone, n(%)						< 0.001
No	18,905(98.35%)	4690 (97.65%)	4728 (98.34%)	4725 (98.62%)	4762 (98.78%)	
Yes	318(1.65%)	113 (2.35%)	80 (1.66%)	66 (1.38%)	59 (1.22%)	
Carbapenem, n(%)						< 0.001
No	15,511(80.69%)	3589 (74.72%)	3885 (80.80%)	3943 (82.30%)	4094 (84.92%)	
Yes	3712(19.31%)	1214 (25.28%)	923 (19.20%)	848 (17.70%)	727 (15.08%)	
Cephalosporins, n(%)						0.088
No	17,775(92.47%)	4472 (93.11%)	4427 (92.08%)	4404 (91.92%)	4472 (92.76%)	
Yes	1448(7.53%)	331 (6.89%)	381 (7.92%)	387 (8.08%)	349 (7.24%)	
Penicillins, n(%)						< 0.001
No	10,026(52.16%)	2206 (45.93%)	2531 (52.64%)	2654 (55.40%)	2635 (54.66%)	
Yes	9197(47.84%)	2597 (54.07%)	2277 (47.36%)	2137 (44.60%)	2186 (45.34%)	
Vancomycin, n (%)						< 0.001
No	3648(18.98%)	491 (10.22%)	876 (18.22%)	1018 (21.25%)	1263 (26.20%)	
Yes	15,575(81.02%)	4312 (89.78%)	3932 (81.78%)	3773 (78.75%)	3558 (73.80%)	
28-day mortality						< 0.001
No	15,545(80.87%)	3403 (70.85%)	3976 (82.70%)	4077 (85.10%)	4089 (84.82%)	
Yes	3678(19.13%)	1400 (29.15%)	832 (17.30%)	714 (14.90%)	732 (15.18%)	

Variables are presented as mean ± SD, median (IQR) or N (%)

*SOFA* Sequential Organ Failure Assessment, *IVIG* Intravenous immunoglobulin, *MV* Mechanical ventilation, *MAP* Mean arterial Pressure, *RRT* Rrenal replacement therapy

## Result

### Patient screening process description

**Fig. 1:**
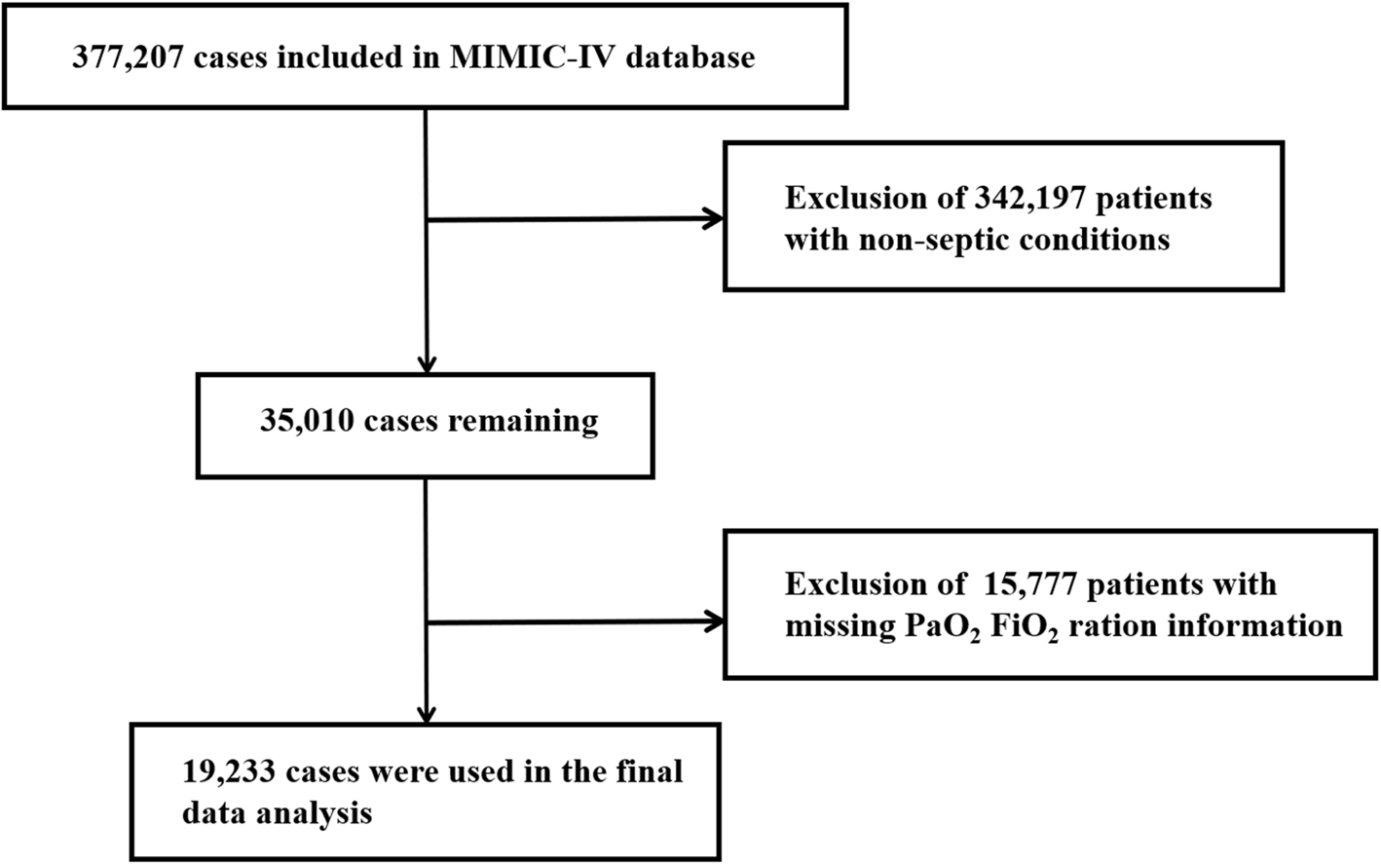
Flow chart. MIMIC: Medical Information Mart for Intensive Care

A total of 377, 207 cases from the MIMIC-IV database were enrolled in the study. There were 342, 297 non-septic patients and 15, 777 patients with missing PaO_2_/FiO_2_ information. Therefore, 19, 233 cases were included in the final analysis. The patient selection flow chart is shown in Fig. 1.

### Baseline characteristics of patients

The baseline characteristics of patients are listed in Table 1. Based on the quartile grouping, LnPaO_2_/FiO_2_ of the overall population was equally divided into four groups (Q1 to Q4). Then the characteristics of the distribution of each variable in each group were analyzed. The mean age of patients was 65.57 ± 15.65 years. The 28-day mortality rate in patients with sepsis was 19.13% (3678/19, 233). The distribution of cephalosporin antibiotic did not differ statistically significantly in different LnPaO_2_/FiO_2_ subgroups (*p* = 0.088). Compared with high-level (Q4) of LnPaO_2_/FiO_2_ group, patients had higher age, higher Charlson Comorbidity Index, SOFA scores, body temperatures, respiratory and heart rate, higher FiO_2_, and had greater percentages of using RRT, dopamine, dobutamine, noradrenaline, methylprednisolone, cortisone, intravenous immunoglobulin, carbapenem, penicillin, and vancomycin antibiotics in other three groups (Q1 ~ Q3). In contrast, higher MAP and PaO_2_, lower rates of using dexamethasone and mechanical ventilation in Q4 group was observed compared with other three groups (Q1 ~ Q3).

### The relationship between LnPaO_2_/FiO_2_ and 28-day mortality in patients with sepsis using non-adjusted and adjusted models

Different covariate adjustment strategies were used to enlighten the association between LnPaO_2_/FiO_2_ and 28-day mortality in patients with sepsis. The non-adjusted and adjusted models are shown in Table 2. In non-adjusted model, for each 1 increase in LnPaO_2_/FiO_2_, the risk of 28-day death in patients with sepsis was decreased by 51% (OR: 0.49, 95%CI: 0.46—0.52). In adjusted I model (sex, age at admission and ethnicity were adjusted), the trend of OR did not to be altered (OR: 0.49, 95%CI: 0.46—0.52, *p* < 0.001). In adjusted II model (sex, age at admission, ethnicity, Charlson Comorbidity Index, SOFA scores, the use of dexamethasone, methylprednisolone, cortisone, noradrenaline, dopamine, dobutamine, IVIG, the use of mechanical ventilation and RRT, the use of carbapenem, cephalosporins, penicillin, vancomycin, heart rate, respiratory rate, temperature and MAP were adjusted), the risk of 28-day death was decreased by 28% (OR: 0.72, 95%CI: 0.67—0.79, *p* < 0.001). For sensitivity analysis, we also handled LnPaO_2_/FiO_2_ as a categorical variable (Quartile). The same trend was observed as well (*p* for trend was 0.0005). The Hosmer–Lemeshow Test shown that the three models were not a good fit and further fitting with the GAM model was required.

**Table 2:** Relationship between LnPaO_2_/FiO_2_ and 28-day mortality in patients with sepsis

Exposure	Non-adjusted (OR, 95%CI, *p*)	Adjust I (OR, 95%CI, *p*)	Adjust II (OR, 95%CI, *p*)
LnPaO_2_/FiO_2_	0.49 (0.46, 0.52) < 0.0001	0.49 (0.46, 0.52) < 0.0001	0.72 (0.67,0.79) < 0.0001
LnPaO_2_/FiO_2_(quartile)			
Q1	Ref	Ref	Ref
Q2	0.51 (0.46, 0.56) < 0.0001	0.50 (0.45, 0.55) < 0.0001	0.60 (0.53, 0.67) < 0.0001
Q3	0.43 (0.38, 0.47) < 0.0001	0.41 (0.37, 0.46) < 0.0001	0.66 (0.59, 0.75) < 0.0001
Q4	0.44 (0.39, 0.48) < 0.0001	0.44 (0.40, 0.49) < 0.0001	0.82 (0.72, 0.93) 0.0017
*P* for trend	< 0.0001	< 0.0001	0.0005
Chi-square / *P* for H–L Test	81.033 / < 0.001	37.862 / < 0.001	20.337 / 0.009

Non-adjusted model adjusted for: None

Adjust I model adjusted for: sex, age at admission, ethnicity

Adjust II model adjusted for: sex, age at admission, ethnicity, Charlson Comorbidity Index, SOFA scores, the use of dexamethasone, methylprednisolone, cortisone, noradrenaline, dopamine, dobutamine, IVIG, the use of mechanical ventilation and RRT, the use of carbapenem, cephalosporins, penicillin, vancomycin, heart rate, respiratory rate, temperature and MAP

*OR* Odds ratio, *CI* Confidence interval, *Ref* Rreference, *H–L Test* Hosmer Lemeshow Test

### Non-linear relationship between LnPaO_2_/FiO_2_ and 28-day mortality in patients with sepsis

**Fig. 2:**
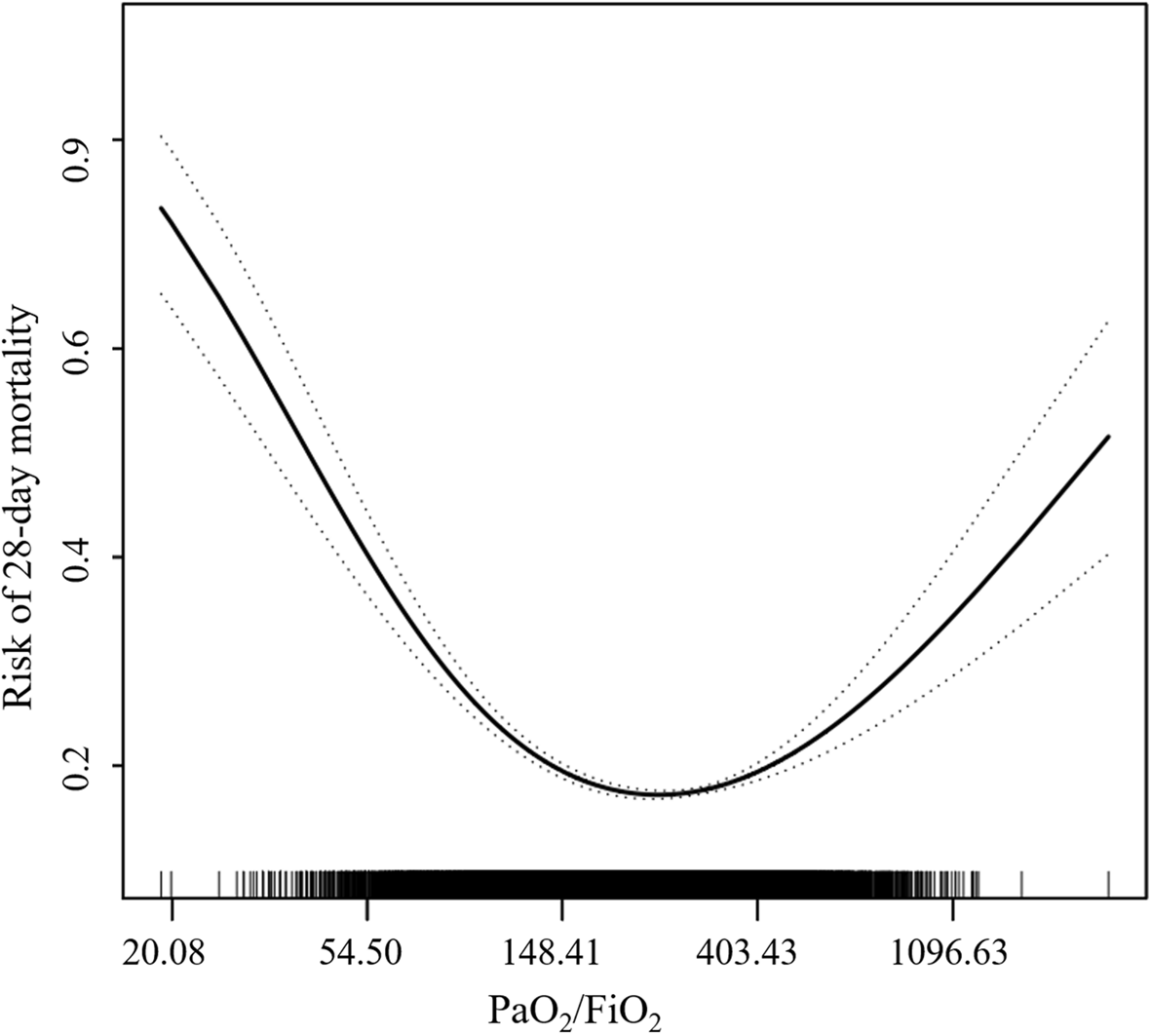
The non-linear relationship between PaO_2_/FiO_2_ and 28-day mortality in patients with sepsis

We explored the non-linear relationship between LnPaO_2_/FiO_2_ and 28-day mortality in patients with sepsis using generalized additive model and smoothed curve fitting. We found that the relationship between LnPaO_2_/FiO_2_ and 28-day mortality in patients with sepsis was U-shape (sex, age at admission, ethnicity, Charlson Comorbidity Index, SOFA scores, the use of dexamethasone, methylprednisolone, cortisone, noradrenaline, dopamine, dobutamine, IVIG, the use of mechanical ventilation and RRT, the use of carbapenem, cephalosporins, penicillin, vancomycin, heart rate, respiratory rate, temperature and MAP were adjusted). For ease of comprehension of the results, calculated after conversion, the non-linear relationship between PaO_2_/FiO_2_ and 28-day mortality in patients with sepsis is shown in Fig. 2. By two-piecewise linear regression model and recursive algorithms, the inflection point of LnPaO_2_/FiO_2_ was 5.30 (95%CI: 5.21—5.39). Calculated after conversion, i.e., the inflection point of PaO_2_/FiO_2_ was 200.33 mmHg (95%CI: 183.09—219.20 mmHg). On the left of inflection point, for each 1 increase in LnPaO_2_/FiO_2_ (or a 2.72 mmHg increases in PaO_2_/FiO_2_), the risk of sepsis 28-day death was decreased by 63% (OR: 0.37, 95%CI: 0.32—0.43, *p* < 0.0001). On the right of inflection point, for each 1 increase in LnPaO_2_/FiO_2_, the risk of sepsis 28-day death was increased 53% (OR: 1.53, 95%CI: 1.31—1.80, *p* < 0.0001) (Table 3).

**Table 3:** Threshold effect analysis for the relationship between LnPaO_2_/FiO_2_ and 28-day mortality in patients with sepsis

**Outcome:**	OR (95% CI)	*P*-value
Fitting model using standard logistic regression model	0.72 (0.68, 0.81)	< 0.0001
Fitting model using two-piecewise linear model		
5.30	0.37 (0.32, 0.43)	< 0.0001
≥ 5.30	1.53 (1.31, 1.80)	< 0.0001
Log-likelihood ratio test		< 0.001

ORs were adjusted for: sex, age at admission, ethnicity, Charlson Comorbidity Index, SOFA scores, the use of dexamethasone, methylprednisolone, cortisone, noradrenaline, dopamine, dobutamine, IVIG, the use of mechanical ventilation and RRT, the use of carbapenem, cephalosporins, penicillin, vancomycin, heart rate, respiratory rate, temperature and MAP

## Discussion

Based on 19, 233 sepsis patients in the MIMIC-IV database, this large retrospective study found that the PaO_2_/FiO_2_ was independently associated with a 28-day mortality in patients with sepsis. After covariate adjustment strategies and sensitivity analysis, a U-shape relationship between PaO_2_/FiO_2_ and 28-day mortality was revealed. The results indicated that either a high or low PaO_2_/FiO_2_ was associated with an increased risk of death in sepsis patients. Moreover, we found that PaO_2_/FiO_2_ between 183.09 mmHg and 219.20 mmHg was associated with a lower risk of 28-day death in patients with sepsis.

As mentioned before, PaO_2_/FiO_2_ as one of items in SOFA score could reflect the severity of illness. Some studies have shown that PaO_2_/FiO_2_ was an independent risk factor for 28-day death in patients with sepsis [9, 10, 20], which was also confirmed our study. However, there was a U-shape relationship between PaO_2_/FiO_2_ and 28-day mortality in patients with sepsis through analysis using generalized additive model and smoothed curve fitting.

Apart from FiO_2_ values, it was also a U-shaped association between PaO_2_ during the first 24 h after ICU admission in mechanically ventilated patient [21]. As we know, sepsis is a major disease in ICU. A retrospective study conducted by Zhongheng Zhang and colleagues, which used data from the MIMIC-II database and included 11, 002 ICU patients, showed that the relationship between PaO_2_ levels and mortality in sepsis patients was quadratic and non-linear [22]. PaO_2_ is usually affected by FiO_2_, therefore we used PaO_2_/FiO_2_ to explore the association between hypoxaemia and 28-day mortality. These studies indicate that patients with either a very low or high PO_2_ have a higher mortality rate. Low PaO_2_ in patients means hypoxaemia, which related to anaerobic metabolism, cellular dysfunction, and progressive metabolic lactic acidosis. High PO_2_ in patients is not good either, which leads to pulmonary toxicity, augmented ischemia–reperfusion injury, and systemic vasoconstriction with decreased organ perfusion [23, 24]. In this study, we used the updated MIMIC database, and the exposure variables were composite indicators and more abundant. The adjustment strategy was focused on adjusting treatment (such as the use of dexamethasone, methylprednisolone, cortisone, the use of noradrenaline, dopamine, dobutamine, IVIG, the use of mechanical ventilation and RRT, the use of carbapenem, cephalosporins, penicillin, vancomycin).

In addition, we found that PaO_2_/FiO_2_ in the range of 183.09 mmHg to 219.20 mmHg, was associated with a lower risk of death in patients with sepsis. This result is consistent with a previous study conducted by Peng et al. The study also used the MIMIC-IV database. Machine learning was used to identify sepsis subphenotypes and compare the clinical outcomes for subphenotypes. The PaO_2_/FiO_2_ in subphenotype A and B were 202 (130—285) mmHg *vs* 113 (74—183) mmHg respectively (*p* < 0.001) [25]. Mean PaO_2_/FiO_2_ for subphenotype A patients similar our threshold period. The researchers found that the hospital mortality in participants with subphenotype B was higher than subphenotype A. Why does a higher PaO_2_/FiO_2_ increase patient mortality? This might be attributable to the fact that the relation of the PaO_2_/FiO_2_ as a function of the FiO_2_ is non-linear and can be U-shaped depending on the underlying shunt fraction and the arterial-mixed venous oxygen content function [26]. Consequently, the PaO_2_/FiO_2_ can increase sharply at very high FiO_2_. Sustained exposure to FiO_2_ of 0.7 or greater was toxic across numerous species. HYPERS2S trail showed setting FiO_2_ to 1.0 to induce arterial hyperoxia might increase the risk of mortality in patients with septic shock [27]. This is mainly related to excessive production of reactive oxygen species (ROS) [23].

### Strengths and limitations

Firstly, the large sample size provides us with more reliable results, allowing us to better understand the association between PaO_2_/FiO_2_ and 28-day mortality in patients with sepsis. Secondly, sensitivity analysis and non-linear algorithm used in this study can help us better observe and address the association between PaO_2_/FiO_2_ and 28-day mortality in patients with sepsis. However, this study has the following limitations. Firstly, the study's population was primarily from the United States, so additional clinical studies were required to determine whether the findings can be applied to populations from other countries. Secondly, as this was an observational study, confounding could not be avoided. Although, we rigorously adjusted for confounding and used sensitivity analysis to assess the robustness of the results. Thirdly, due to the limitations of observational studies, we can only observe associations and cannot assess cause and effect. Fourthly, although there were some antibiotics were chosen as variables, some may still be ignored. Fifthly, considering the logistic regression fit was powerless, the binary logistic regression results should be regarded with caution. Lastly, we could only adjust for measurable confounding, not non-measurable confounding, implying that larger population clinical studies with higher levels of evidence may be required to validate our findings.

## Conclusion

There was a U-shaped relationship between PaO_2_/FiO_2_ and 28-day mortality in patients with sepsis. In the range of 184.93 mmHg to 219.20 mmHg, PaO_2_/FiO_2_ was associated with a lower risk of death in patients with sepsis. This finding indicates that we should pay more attention to PaO_2_/FiO_2_ levels in clinical work.

## Data Availability

The datasets analyse during the current study are available in the MIMIC-IV repository, https://physionet.org/content/mimiciv/0.4/. The links is the direct persistent links to the datasets and researchers need to completed the course Protecting Human Research Participants on the website of National Institutes of Health and obtained the certification prior to accession. The data can be accessed from the corresponding author Xu Liu, e-mail: 262347762@qq.com.

## Abbreviations

ICU: Intensive care unit
ARDS: Acute respiratory distress syndrome
SOFA: Sequential Organ Failure Assessment
ML: Machine learning
MIMIC-IV: Medical information marketplace for intensive care IV
GAM: Generalized additive mode
IVIG: Intravenous immunoglobulin
MV: Mechanical ventilation
OR: Odds ratio
CI: Confidence interval
Ref: Reference
MAP: Mean arterial pressure
RRT: Renal replacement therapy
